# Etanercept treatment for extended oligoarticular juvenile idiopathic arthritis, enthesitis-related arthritis, or psoriatic arthritis: 6-year efficacy and safety data from an open-label trial

**DOI:** 10.1186/s13075-019-1916-9

**Published:** 2019-05-23

**Authors:** Ivan Foeldvari, Tamàs Constantin, Jelena Vojinović, Gerd Horneff, Vyacheslav Chasnyk, Joke Dehoorne, Violeta Panaviene, Gordana Sušić, Valda Stanevicha, Katarzyna Kobusinska, Zbigniew Zuber, Bogna Dobrzyniecka, Irina Nikishina, Brigitte Bader-Meunier, Luciana Breda, Pavla Doležalová, Chantal Job-Deslandre, Ingrida Rumba-Rozenfelde, Nico Wulffraat, Ronald D. Pedersen, Jack F. Bukowski, Bonnie Vlahos, Alberto Martini, Nicolino Ruperto, for the Paediatric Rheumatology International Trials Organisation (PRINTO)

**Affiliations:** 1 Hamburg Centre for Pediatric and Adolescent Rheumatology, Hamburg, Germany; 2 Unit of Pediatric Rheumatology-Immunology, Second Department of Pediatrics, Semmelweis University, Budapest, Hungary; 3 Clinic of Pediatrics, Clinical Center Niš, Faculty of Medicine, University of Niš, Niš, Serbia; 4 Department of General Paediatrics, Asklepios Clinic Sankt Augustin, Sankt Augustin, Germany; 5 Department of Paediatric and Adolescents Medicine, Medical Faculty, University Hospital of Cologne, Cologne, Germany; 6 Saint-Petersburg State Pediatric Medical University, Saint-Petersburg, Russian Federation; 7 Department of Pediatric Rheumatology, Ghent University Hospital, Ghent, Belgium; 8 Children’s Hospital, Affiliate of Vilnius University Hospital Santaros Clinic, Vilnius, Lithuania; 9 Clinic of Children’s Diseases, Vilnius University, Vilnius, Lithuania; 10 Division of Pediatric Rheumatology, Institute of Rheumatology, Belgrade, Serbia; 11 Department of Pediatrics, Riga Stradins University, Children University Hospital, Riga, Latvia; 12 Provincial Children’s Hospital J. Brudzińskiego, Bydgoszcz, Poland; 13 Andrzej Frycz Modrzewski Krakow University, Krakow, Poland; 14 Szpital Specjalistyczny im. A. Falkiewicza, Szpital Specjalistyczny, Wroclaw, Poland; 15 Pediatric Department, V.A. Nasonova Research Institute of Rheumatology, Moscow, Russian Federation; 16 IMAGINE Institute, Hôpital Necker-Enfants Malades, Centre de Référence National pour les Rhumatismes Inflammatoires et les Maladies Auto-Immunes Sytémiques rares de l’enfant (RAISE), Unité d’Immunologie, Hématologie et Rhumatologie Pediatrique, Paris, France; 17 Dipartimento di Pediatria, Ospedale Policlinico – Università degli Studi di Chieti, Chieti, Italy; 18 Department of Pediatrics and Adolescent Medicine, General University Hospital and 1st Faculty of Medicine, Charles University in Prague, Prague, Czech Republic; 19 Hôpital Universitaire Cochin, Centre de Reference National pour les Arthrites Juveniles, Site Patients Adultes - Service Rhumatologie A, Paris, France; 20 Faculty of Medicine, University of Latvia, Riga, Latvia; 21 University Children Hospital, Riga, Latvia; 22 Department of Pediatric Immunology and Rheumatology, Wilhelmina Children’s Hospital, Utrecht, The Netherlands; 23 Pfizer, Collegeville, PA USA; 24 Dipartimento di Neuroscienze, Riabilitazione, Oftalmologia, Genetica e Scienze Materno-Infantili (DiNOGMI), Università degli Studi di Genova, Genoa, Italy; 25 Clinica Pediatrica e Reumatologia, IRCCS Istituto Giannina Gaslini, EULAR Centre of Excellence in Rheumatology 2008-2023, Paediatric Rheumatology International Trials Organisation (PRINTO), Via Gaslini, 5, 16147 Genoa, Italy

**Keywords:** Etanercept, Juvenile idiopathic arthritis, Enthesitis-related arthritis, Extended oligoarticular juvenile idiopathic arthritis (eoJIA), Enthesitis-related arthritis (ERA), Psoriatic arthritis (PsA), Efficacy, Safety, Clinical trial

## Abstract

**Background:**

To describe the 6-year safety and efficacy of etanercept (ETN) in children with extended oligoarticular juvenile idiopathic arthritis (eoJIA), enthesitis-related arthritis (ERA), and psoriatic arthritis (PsA)

**Methods:**

Patients who completed the 2-year, open-label, phase III *CL*inical Study *I*n *P*ediatric *P*atients of *E*tanercept for Treatment of E*R*A, PsA, and Extended Oligoarthritis (CLIPPER) were allowed to enroll in its 8-year long-term extension (CLIPPER2). Children received ETN at a once-weekly dose of 0.8 mg/kg, up to a maximum dose of 50 mg/week. Efficacy assessments included the JIA core set of outcomes, the JIA American College of Rheumatology response criteria (JIA-ACR), and the Juvenile Arthritis Disease Activity Score (JADAS). Efficacy data are reported as responder analyses using a hybrid method for missing data imputation and as observed cases. Safety assessments included treatment-emergent adverse events (TEAEs).

**Results:**

Out of 127 patients originally enrolled in CLIPPER, 109 (86%) entered CLIPPER2. After 6 years of trial participation (2 years in CLIPPER and 4 years in CLIPPER2), 41 (32%) patients were still taking ETN, 13 (11%) entered the treatment withdrawal phase after achieving low/inactive disease (of whom 7 had to restart ETN), 36 (28%) discontinued treatment for other reasons but are still being observed, and 37 (29%) discontinued treatment permanently. According to the hybrid imputation analysis, proportions of patients achieving JIA ACR90, JIA ACR100, and JADAS inactive disease after the initial 2 years of treatment were 58%, 48%, and 32%, respectively. After the additional 4 years, those proportions in patients who remained in the trial were 46%, 35%, and 24%. Most frequently reported TEAEs [*n* (%), events per 100 patient-years] were headache [28 (22%), 5.3], arthralgia [24 (19%), 4.6], and pyrexia [20 (16%), 3.8]. Number and frequency of TEAEs, excluding infections and injection site reactions, decreased over the 6-year period from 193 and 173.8, respectively, during year 1 to 37 and 61.3 during year 6. A single case of malignancy (Hodgkin’s lymphoma) and no cases of active tuberculosis, demyelinating disorders, or deaths were reported.

**Conclusions:**

Open-label etanercept treatment for up to 6 years was safe, well tolerated, and effective in patients with eoJIA, ERA, and PsA.

**Trial registration:**

ClinicalTrials.gov: CLIPPER, NCT00962741, registered 20 August, 2009, CLIPPER2, NCT01421069, registered 22 August, 2011.

**Electronic supplementary material:**

The online version of this article (10.1186/s13075-019-1916-9) contains supplementary material, which is available to authorized users.

## Background

Juvenile idiopathic arthritis (JIA) is a heterogeneous chronic disease estimated to affect approximately 1 in 1000 children [1–3]. It encompasses seven clinical categories: systemic arthritis, oligoarticular juvenile idiopathic arthritis (extended and persistent: eoJIA and poJIA), rheumatoid factor-positive polyarthritis, rheumatoid factor-negative polyarthritis, psoriatic arthritis (PsA), enthesitis-related arthritis (ERA), and undifferentiated arthritis [4]. Available treatments have greatly improved clinical outcomes [5–11], but few have been studied in all JIA categories [5, 12, 13], with long-term data being relatively scarce [14].

Etanercept (ETN), an inhibitor of tumor necrosis factor alpha (TNFα), was shown to be safe and efficacious in children with polyarticular JIA who received up to 8 years of continuous treatment [15, 16], but evidence of long-term benefits in the JIA categories of eoJIA, ERA, and psoriatic arthritis has been limited [17, 18].

*CL*inical Study *I*n *P*ediatric *P*atients of *E*tanercept for Treatment of E*R*A, PsA, and Extended Oligoarthritis (CLIPPER, NCT00962741) was a 2-year open-label study, designed to assess efficacy and safety of ETN in pediatric patients with eoJIA, ERA, and PsA [19]. CLIPPER and CLIPPER2 (NCT01421069), its 8-year long-term extension, will provide efficacy and safety data for up to 10 years of treatment in this patient population. Here we present 6-year interim findings, from 2 years of CLIPPER and 4 years of CLIPPER2, on safety and efficacy in patients with eoJIA, ERA, or PsA.

## Methods

### Patients and study design

Full methodology of the CLIPPER trial was described previously [8, 19]. Briefly, CLIPPER was a 24-month, Phase IIIb, open-label multicenter study performed at 38 centers in 19 member countries of the Paediatric Rheumatology International Trials Organisation (PRINTO) [20]. Patients classified as eoJIA (2–17 years of age), ERA (12–17 years), or PsA (12–17 years) received ETN 0.8 mg/kg (maximum dose, 50 mg) once weekly (QW) for up to 96 weeks. All participants were required to have ≥ 2 active joints (e.g., joints with swelling or with limitation of motion [LOM] accompanied by either pain or tenderness). Those with eoJIA or PsA were required to have had an unsatisfactory response or intolerance to a non-biologic disease-modifying anti-rheumatic drug (DMARD) (e.g., methotrexate). Patients with ERA were required to have had an unsatisfactory response or intolerance to either a non-biologic DMARD or a non-steroidal anti-inflammatory drug (NSAID). Individuals previously treated with biologics were excluded. CLIPPER2 is an on-going, 8-year, open-label extension study of CLIPPER. Patients who received at least one dose of ETN and who completed approximately 2 years of CLIPPER were eligible to enter the active treatment period of CLIPPER2.

**Fig. 1:**
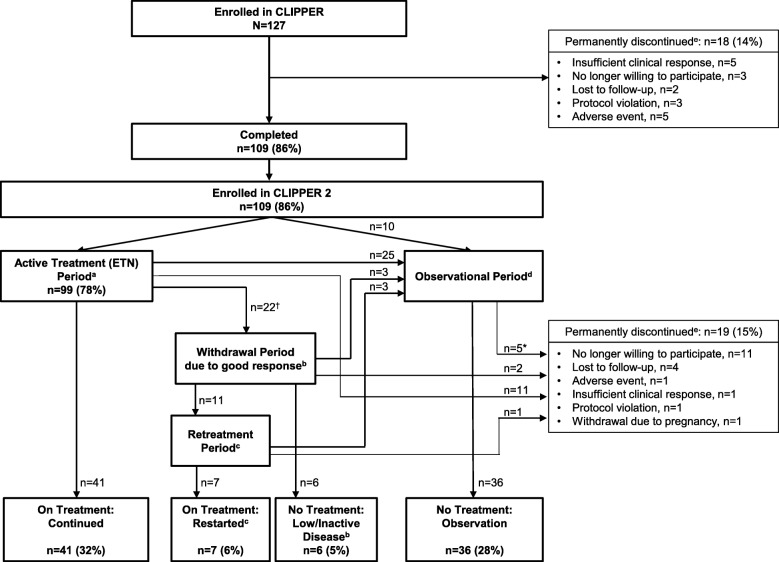
Study flow. (a) Patients actively receiving treatment with ETN. (b) Patients who either achieved CR_ACR_ or who, in the investigator’s clinical judgment, had a good clinical response and would benefit from treatment withdrawal. (c) Patients in the withdrawal period who required re-treatment per the investigator’s clinical judgment and re-started ETN. (d) Patients who stopped treatment but were still followed in CLIPPER2. (e) Patients who were no longer being followed as part of CLIPPER or CLIPPER2. *Includes two patients who entered the observational period directly from CLIPPER, plus three patients who entered the observational period from another treatment phase. ^†^Nine out of 22 patients entered the withdrawal period because of a treatment-emergent adverse event. Abbreviations: CR_ACR_, clinical remission, based on the American College of Rheumatology criteria [21]

A combined flow chart of CLIPPER and CLIPPER2 is presented in Fig. 1. All CLIPPER participants who completed 24 months of treatment with ETN were eligible to enroll in CLIPPER2. Patients who had either met the American College of Rheumatology (ACR) definition for JIA clinically inactive disease [21] (CID_ACR_) for at least 6 months of continuous treatment (clinical remission, CR_ACR_) or, in investigator’s judgment, had a good clinical response and would benefit from etanercept withdrawal, were eligible to enter the withdrawal period. Those in the withdrawal period who flared and were in need of ETN retreatment per the investigator’s clinical judgment could enter the retreatment period. (flare was defined as ≥ 30% worsening in at least three of the six ACR Pediatric components, with ≥ 30% improvement in not more than one of the remaining components and a minimum of two active joints.) Patients who did not complete 24 months of active treatment in CLIPPER or discontinued ETN for any reason before the end of CLIPPER2, as well as those in the withdrawal period who were ineligible for retreatment with ETN or who discontinued during the retreatment period, were eligible to enroll into the observational period of CLIPPER2 and were assessed for safety only, every 6 months until the end of the trial.

### Efficacy and safety outcomes

The efficacy endpoints were assessed for up to 72 months of trial participation (24 months of CLIPPER and 48 months of CLIPPER2) and included the JIA core set of measures [22]: Physician Global Assessment (PGA) [23], Patient/Parent Global Assessment (PtGA) [23], number of active joints and joints with LOM, levels of C-reactive protein (CRP), and the cross-culturally adapted and validated version of Childhood Health Assessment Questionnaire (CHAQ) [24]. In addition, we reported the overall back pain and nocturnal back pain on a 100-mm visual analog scale (VAS) for patients with ERA; body surface area (BSA; %) affected and PGA of psoriasis for patients with PsA; proportions of patients achieving JIA American College of Rheumatology’s (ACR) 30, 50, 70, 90, and 100 criteria [21], and the Juvenile Arthritis Disease Activity Score (JADAS) [25] 73-joint inactive disease, JADAS clinically inactive disease (CID_JADAS_) [26] at each visit; and proportions of patients achieving sustained JIA ACR clinical remission (CR_ACR_) defined as no joints with active arthritis; no fever, rash, serositis, splenomegaly, or generalized lymphadenopathy attributable to JIA; CRP level within normal limits; PGA of disease activity score of best possible on the scale used; duration of morning stiffness of ≤ 15 min; and no active uveitis [21]. CR on medication was defined as persistence of clinically inactive disease (CR_ACR_ or CR_JADAS_ criteria) for ≥ 6 continuous months. Disease activity was assessed based on the following cut-off values of the JADAS score: ≤ 1, CID; 1–3.8, low disease activity (LDA); 3.9–10.5, moderate disease activity (MDA); > 10.5, high disease activity (HDA) [25–27]. Time to disease flare was assessed for patients who entered the withdrawal period.

Safety outcomes included reports of infections, malignancy, and other treatment-emergent adverse events (TEAEs) classified as per the latest release of MedDRA.

### Statistical analysis

All efficacy and safety analyses were based on the modified intention-to-treat (mITT) population, defined as all patients who received at least one dose of ETN.

For the responder analyses, missing values were imputed using a hybrid method, based on patients’ enrolment status, trial period at cut-off date, and reasons for permanent discontinuation (Additional file 1: Table S1). Specifically, for patients who did not complete CLIPPER, missing data were considered non-response (non-responder imputation, NRI). For those who did complete CLIPPER but did not enroll in CLIPPER2, imputation was performed using the last observation carried forward (LOCF) approach. For patients enrolled in CLIPPER2 and who were in active treatment period at the cut-off date, observed cases (OC) were used (i.e., there was no imputation). For those enrolled in CLIPPER2, patients who were in the withdrawal or retreatment period at the cut-off date, or who permanently discontinued the trial for pregnancy-related reasons, the LOCF approach was used, using the last available data from the active treatment period. For patients enrolled in CLIPPER who were in the observational period at the cut-off date, or who permanently discontinued treatment for reasons not related to pregnancy, the NRI approach was used. Finally, for those who enrolled in CLIPPER2 and had missing values before the protocol amendment that added efficacy assessments, those who entered the observational period directly, entered the withdrawal period or dropped out for reasons related to pregnancy, or had not entered the withdrawal period and had no efficacy data at the time of cut-off, the LOCF approach was used. Supporting responder analyses were conducted using OC and the more conservative NRI approaches (Additional file 1: Figure S1). Data are presented as means or proportions (%), with 95% confidence intervals.

All other efficacy analyses were conducted using the OC approach. Median time to flare for patients in the withdrawal period was determined using Kaplan-Maier analysis.

TEAEs were summarized as numbers of events (*n*) and events per 100 patient-years (EP100PY). For patients in the observational period of CLIPPER2, safety was assessed by collecting serious AEs only.

## Results

### Patient disposition and baseline characteristics

Six years after treatment initiation (i.e., after 2 years of CLIPPER and 4 years of CLIPPER2), 48 (38%) of patients enrolled in CLIPPER (*N* = 127) were still receiving etanercept, either continuously (41 [32%]) or as retreatment (7 [6%]), whereas 6 (5%) stopped treatment due to low/inactive disease and 36 (28%) because they entered the observation period during CLIPPER2 (Fig. 1). A total of 37 (29%) patients permanently discontinued trial participation: 18 (14%) during CLIPPER and 19 (15%) during CLIPPER2 (Fig. 1). Additional 36 (28%) stopped ETN treatment but remained in the observational period, for a total of 73 (46%) patients who discontinued the study drug.

Demographic and clinical characteristics of patients entering CLIPPER2 (Table 1) were similar to the population enrolled in CLIPPER, which were described previously [8]. The proportions of patients with PsA and ERA who entered CLIPPER2 (79% and 82%, respectively) were lower than the proportion of patients with eoJIA (92%).

**Table 1:** Baseline characteristics at enrollment in the open-label extension phase, after 24 months of treatment with etanercept (baseline CLIPPER2)

	eoJIA *n* = 55	ERA *n* = 31	PsA *n* = 23	Total *n* = 109
% of CLIPPER	92% (55/60)	82% (31/38)	79% (23/29)	86% (109/127)
Age, years^a^	10.6 (4.6)	16.2 (1.6)	15.8 (2.4)	13.3 (4.5)
Female^a^	38 (69)	5 (16)	18 (78)	61 (56)
JIA core set				
Physician Global Assessment of disease activity, 0–100 score	1.0 (1.4) *n* = 49	0.7 (0.7) *n* = 30	0.8 (1.0) *n* = 19	0.8 (1.2) *n* = 98
Number of active joints^a^	0.6 (1.0) *n* = 40	0.7 (1.2) *n =* 28	1.3 (4.6) *n =* 19	0.8 (2.3) *n* = 87
Number of joints with LOM^a^	0.8 (1.1) *n =* 40	1.4 (3.2) *n =* 28	1.7 (5.0) *n =* 19	1.2 (3.0) *n =* 87
C-reactive protein, mg/L^a^ (normal < 5 mg/L)	3.9 (8.5) *n* = 47	2.7 (4.1) *n* = 27	1.1 (0.2) *n* = 18	3.0 (6.5) *n* = 92
PtGA score^a^	1.4 (2.0)	1.0 (1.3)	1.3 (1.5)	1.3 (1.7)
CHAQ score^a^	0.3 (0.6) *n =* 47	0.1 (0.2) *n =* 20	0.2 (0.3) *n* = 13	0.2 (0.5) *n* = 80
JADAS 73 score^a^	3.5 (4.7) *n* = 37	2.3 (2.2) *n* = 25	3.3 (5.4) *n =* 18	3.1 (4.3) *n =* 80
Additional measures				
Overall back pain VAS, mm^a^	– *n* = 0	2.3 (4.8) *n* = 26	– *n =* 0	2.3 (4.8) *n =* 26
Nocturnal back pain VAS, mm^a^	– *n =* 0	2.1 (3.7) *n =* 26	– *n =* 0	2.1 (3.7) *n =* 26
Psoriasis BSA, percentage^a^	– *n =* 0	– *n =* 0	1.4 (2.4) *n =* 19	1.4 (2.4) *n =* 19
PGA of psoriasis^a^	– *n =* 0	– *n =* 0	0.6 (0.9) *n =* 19	0.6 (0.9) *n =* 19
Baseline therapies^b^				
Any DMARD	50 (91)	28 (90)	19 (83)	97 (89)
Methotrexate	45 (82)	17 (55)	16 (70)	78 (72)
Oral corticosteroid	7 (13)	7 (23)	1 (4)	15 (14)
Oral NSAID	26 (47)	19 (61)	9 (39)	54 (50)

^a^Mean (SD)

^b^Number (percentage)

Abbreviations: BSA, body surface area; CHAQ, Childhood Health Assessment Questionnaire; DMARD, disease-modifying anti-rheumatic drug; eoJIA, extended oligoarticular juvenile idiopathic arthritis; ERA, enthesitis-related arthritis; JADAS, Juvenile Arthritis Disease Activity Score; JIA, juvenile idiopathic arthritis; LOM, limitation of motion; NSAID, non-steroid anti-inflammatory drug; PGA, Physician Global Assessment; PtGA, Patient/Parent Global Assessment; PsA, psoriatic arthritis; SD, standard deviation; VAS, visual assessment scale

The median age of patients with eoJIA (8.0 years) was lower than the median age of patients with ERA or PsA (14.0 years for both), which was the consequence of the study’s inclusion criteria. Most patients with eoJIA and PsA were female (69% and 78%, respectively), whereas the majority of patients with ERA were male (84%). After 24 months of etanercept treatment in CLIPPER, there were differences between the three JIA categories in the mean values of JADAS score, CRP, number of active joints, and joints with LOM. Overall, 89% of patients were receiving DMARDs at baseline of CLIPPER2, with methotrexate being the most commonly used (72%) (Table 1).

### Efficacy

**Fig. 2:**
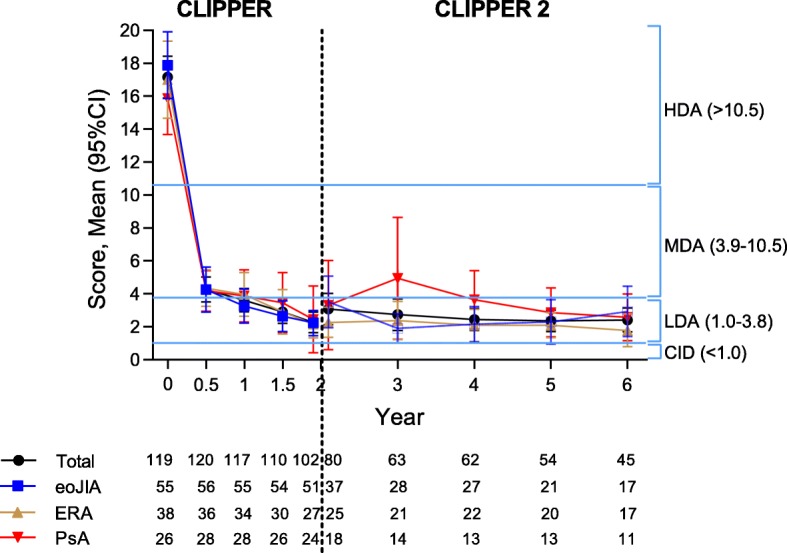
JADAS 73 Score by visit (observed cases). CI, confidence interval; CID, clinically inactive disease; eoJIA, extended oligoarticular juvenile idiopathic arthritis; ERA, enthesitis-related arthritis; HDA, high disease activity; JADAS, Juvenile Arthritis Disease Activity Score; LDA, low disease activity; MDA, moderate disease activity; PsA, psoriatic arthritis

The mean improvements from baseline in JADAS disease activity at month 24 of CLIPPER were largely maintained at month 48 of CLIPPER2 (Fig. 2). A similar pattern was observed for other measures of disease activity, as well as for patient-reported outcomes (Additional file 1: Table S2).

In patients with ERA, improvements in back pain and nocturnal back pain achieved from baseline to month 24 in CLIPPER (reductions in mean score from 25.9 to 2.4 and from 16.4 to 2.2, respectively) were largely maintained during the first 48 months of CLIPPER2; a similar pattern was observed for improvements in BSA and PGA of psoriasis in patients with PsA (Additional file 1: Table S3).

**Fig. 3:**
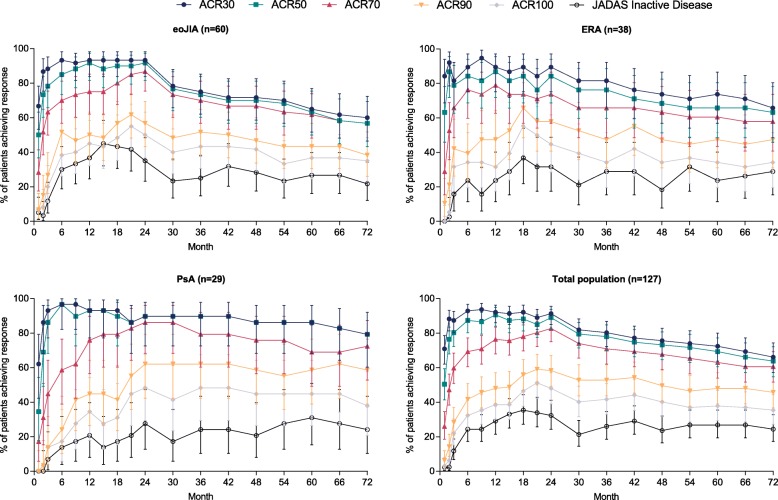
ACR30–100 and JIA inactive disease response rates by visit (hybrid imputation method). Note: For patients with PsA, values for ACR30 and ACR50 overlap completely for all visits after week 24. Abbreviations: eoJIA, extended oligoarticular juvenile idiopathic arthritis; ERA, enthesitis-related arthritis; PsA, psoriatic arthritis

There was a similar pattern in response rates across the three JIA categories obtained using the hybrid method for data imputation, with the strongest initial response in terms of JADAS inactive disease observed for patients with eoJIA (Fig. 3), which was supported by analyses conducted using OC-only and NRI-only methodologies for missing data imputation (Additional file 1: Figure A1).

CR_ACR_ or CR_JADAS_ was achieved by 27% (34/127) and 24% (30/127) of all patients, respectively. After 2 years, there was an apparent overall decline in the proportions of ACR30–100 responders in patients with eoJIA and ERA, but not in those with PsA (Fig. 3).

Twenty-two (17%) patients discontinued ETN treatment due to low activity or inactive disease and entered the withdrawal period (Fig. 1); of those, 13 (59%) experienced disease flares, with a median time to flare of 190 days.

### Safety

Over the 6 years of trials’ duration, the total exposure to ETN amounted to 524.4 patient-years. Exposure and TEAE occurrence data by JIA category are summarized in Table 2.

**Table 2:** Etanercept exposure and TEAEs by JIA category

	eoJIA *n* = 60	ERA *n* = 38	PsA *n* = 29	Total *N* = 127
Exposure, patient-years	245.6	158.9	119.9	524.4
TEAEs (excluding infections and injection site reactions), *n* (*n*/100PY)	244 (99.4)	151 (95.0)	90 (75.0)	485 (92.5)
Infections, *n* (*n*/100PY)	351 (142.9)	93 (58.5)	117 (97.5)	561 (107.0)
TEAEs causing withdrawal (excluding infections and injection site reactions), *n* (*n*/100PY)	5 (2.0)	8 (5.0)	0	13 (2.5)
Infections causing withdrawal, *n* (*n*/100PY)	2 (0.8)	0	1 (0.8)	3 (0.6)
Serious TEAEs, *n* (*n*/100PY)	11 (4.5)	17 (10.7)	4 (3.3)	32 (6.1)
Serious infections, *n* (*n*/100PY)	5 (2.0)	4 (2.5)	4 (3.3)	13 (2.5)
Opportunistic infections^a^, *n* (*n*/100PY)	0	1 (0.6)	1 (0.8)	2 (0.4)
Autoimmune disorders^b^, *n* (*n*/100PY)	4 (1.6)	4 (2.5)	2 (1.7)	10 (1.9)
Malignancies, *n* (*n*/100PY)	1 (0.4)	0	0	1 (0.2)

^a^All opportunistic infections were herpes zoster (recurrent or > 1 dermatome affected)

^b^Uveitis, *n* = 7; Crohn’s disease, *n =* 3

^c^Hodgkin lymphoma

Abbreviations: 100PY, 100 patient-years; eoJIA, extended oligoarticular juvenile idiopathic arthritis; ERA, enthesitis-related arthritis; PsA, psoriatic arthritis; TEAE, treatment-emergent adverse event

The most frequently reported TEAEs after 6 years of etanercept treatment, not including infections and injection site reactions, were headache (*n* = 28; 5.3 EP100PY), arthralgia (*n* = 24; 4.6 EP100PY), pyrexia (*n* = 20; 3.8 EP100PY), diarrhea (*n* = 12; 2.3 EP100PY), and leukopenia (*n* = 12; 2.3 EP100PY). Of those, more than twofold differences in EP100PY by JIA category were observed for headache (eoJIA, 4.9; ERA, 3.2; PsA, 9.2), pyrexia (eoJIA, 4.5; ERA, 1.9; PsA, 3.8), diarrhea (eoJIA, 2.0; ERA, 3.8; PsA, 0.8), and leukopenia (eoJIA, 3.7; ERA, 1.3; PsA, 0.8). Overall, the infections were more common in patients with eoJIA than those with ERA or PsA, with EP1000PY values of 143, 58, and 98, respectively (Table 2).

In the combined 6 years of treatment, there were a total of 561 (107.0 EP100PY) treatment-emergent infections, with the most common being those of the upper respiratory tract (eoJIA *n* = 86, 35.0 EP100PY; ERA 20, 12.6; PsA 34, 28.4), pharyngitis (eoJIA 45, 18.3; ERA 21, 13.2; PsA 20, 16.7), gastroenteritis (eoJIA 18, 7.3; ERA 5, 3.2; PsA 8, 6.7), and bronchitis (eoJIA 16, 6.5; ERA 7, 4.4; PsA 3, 2.5). No individual TEAE occurred more than three times in the same patient. The most frequent TEAEs (> 5% in any JIA subtype) calculated by the number of patients reporting (instead of the number of events and EP100PY) are summarized in Additional file 1: Table S4.

Seven cases of uveitis were reported: three in patients with eoJIA (mild in severity, and judged by the investigator not to be related to treatment), two in patients with ERA (one mild and one moderate in severity, both judged not related to treatment), and two in the PsA subgroup (one mild and one moderate in severity, deemed not related and related to treatment, respectively). There were also four cases of Crohn’s disease: one in a patient with eoJIA (severe, deemed related to treatment) and three in patients with ERA (two moderate and one severe, all deemed not related to treatment). One patient with Crohn’s disease was HLA B27-positive.

A total of 15 patients experienced 19 instances of anemia, leukopenia, or neutropenia (Additional file 1: Table S4). Of those, 12 (80%) were taking methotrexate at baseline, which is comparable with the methotrexate use in the overall trial population (72%; Table 1). One patient (with eoJIA) who was receiving methotrexate entered the withdrawal period because of treatment-emergent leukopenia, but subsequently entered the retreatment period.

A single case of malignancy was reported (Hodgkin lymphoma; Table 2 and Additional file 1: Table S4), in a patient with eoJIA who was treated with ETN for 27 months and had been receiving methotrexate for approximately 8 years. There were no deaths or cases of active tuberculosis or demyelinating disorders.

## Discussion

This is the first report on the safety and efficacy of ETN in patients with eoJIA, ERA, or PsA that spans over 6 years of continuous treatment. The study shows that treatment with ETN is effective, with acceptable safety and tolerability. Disease activity measures and PROs were relatively stable from the previously reported results after 2 years of treatment [19] to the end of year 6 in the current report, suggesting a long-term maintenance of clinical benefits. The maintenance of benefits is particularly evident in the OC analysis (Additional file 1: Figure S1), which, however, included only patients who remained in the study and is therefore biased toward those with a satisfactory response to treatment. An opposite bias is manifest in the NRI analysis, which counts all missing values as non-responders (Additional file 1: Figure S1) and disregards the possibility that patients who discontinued the trial may indeed have had a clinical response. In order to reconcile many possible scenarios for non-response and also account for the fact that 46% of patients discontinued ETN treatment after 6 years, we employed a hybrid method for missing data imputation, which suggests a decline in the response of about 15–20 percentage points between the years 2 and 6, particularly for the more stringent response criteria (ACR50 and above) (Fig. 3). However, about half of this decline was realized during the first 6 months of CLIPPER2, which raises the possibility that it could be attributed, at least partially, to treatment discontinuation, or other administrative differences between two separate clinical trials. Despite this decline, the data demonstrate a substantial long-term effect over a 6-year period.

Direct comparison of our findings with those from previous ETN studies in JIA is difficult, due to differences in time points, response measures, sample size, or JIA disease categories of the patients enrolled [16–18, 28–33]. With these differences in mind, we point out that 61% of patients in our study achieved ACR70 response at 48 months, compared to approximately 75% of patients at 4 years in an open-label extension study of ETN in polyarticular-course JIA [16], and 75% of patients from the Dutch Arthritis and Biologicals in Children Register at 51 months [17]. Similarly, 27% and 24% of patients in our study achieved sustained clinical remission by ACR or JADAS criteria, respectively, compared with 24% (10/42) of patients who had achieved sustained CR_ACR_ (according to the same definition we used in our trial [21]) in a small, 4-year study of children with JIA (predominantly polyarthritis 78%) [34].

Overall, similar rates of TEAEs were observed across all JIA categories and no new major safety signals were observed. However, infections were more common in patients with eoJIA than those with ERA or PsA. Compared with patients with ERA or PsA, the eoJIA subgroup was younger, had a longer disease duration, had higher rates of MTX and CS use, and had a higher disease activity (Table 1) [8], so one or more of these factors may have accounted for the higher infection rate. TEAEs were less frequent after the initial 2 years of treatment. Overall, the TEAE rates were similar to those observed in other long-term studies of ETN in JIA [16, 18, 19, 31].

One patient with eoJIA developed Hodgkin lymphoma after 27 months of treatment with ETN and methotrexate. The incidence of Hodgkin lymphoma in post-marketing ETN data for patients aged 0–17 years was 9.5 per 100,000 patient-years, which is higher than the value for patients in the same age range for the general US population recorded in the Surveillance Epidemiology and End Results database (0.9 per 100,000 patient-years) [35]. However, patients with JIA cannot be easily compared with the general population, since both JIA and the extensive pre-treatment with immuno-suppressants, including methotrexate, have been suggested as additional risk factors for lymphoma [36, 37], and a retrospective study of 2000–2014 US claims data did not find an increased risk of malignancies in TNF-treated children with JIA, pediatric inflammatory bowel disease, or pediatric plaque psoriasis [38]. Nevertheless, a possibility must be allowed that the case of Hodgkin lymphoma observed in our study could have been related to the patient’s treatment (methotrexate, ETN, or both).

The limitations of this study include the non-randomized, open-label design and the relatively low retention of treatment, with many patients with missing data or lost to follow-up. In addition, because of a late protocol amendment, efficacy data are not available for all patients from the beginning of the extension study.

## Conclusions

In conclusion, open-label treatment with ETN up to 6 years was safe, well tolerated, and effective in patients with eoJIA, ERA, and PsA. No new safety signals were detected.

## Additional file

Additional file 1: **Table S1.** Summary of missing data imputation methods for the responder analysis. **Table S2.** Disease activity and patient-reported outcomes (observed cases). **Table S3.** Outcomes specific for enthesitis-related arthritis and psoriatic arthritis (observed cases). **Table S4.** The most frequent TEAEs, excluding infections and injection site reactions (> 5% in any JIA subtype, by System Organ Class). **Figure S1.** ACR30–100 and JIA inactive disease response rates (OC vs NRI). Additional Tables (A1 to A4) and Figure A1 related to missing data imputation based on patients’ enrolment status, trial period at cut-off date, and reasons for permanent discontinuation. (DOCX 224 kb)

## Abbreviations

ACR: American College of Rheumatology
BSA: Body surface area
CHAQ: Childhood Health Assessment Questionnaire
CR: Clinical remission
CRP: C-reactive protein
DMARD: Disease-modifying anti-rheumatic drug
eoJIA: Extended oligoarticular juvenile idiopathic arthritis
EP100PY: Events per 100 patient-years
ERA: Enthesitis-related arthritis
ETN: Etanercept
HDA: High disease activity
JADAS: Juvenile Arthritis Disease Activity Score
LDA: Low disease activity
LOCF: Last observation carried forward
LOM: Limitation of motion
MDA: Moderate disease activity
NSAID: Non-steroid anti-inflammatory drug
PGA: Physician Global Assessment
PsA: Psoriatic arthritis
PtGA: Patient/Parent Global Assessment
SD: Standard deviation
TEAE: Treatment-emergent adverse event
VAS: Visual analog scale
