# Supplementary material for: Etanercept treatment for extended oligoarticular juvenile idiopathic arthritis, enthesitis-related arthritis, or psoriatic arthritis: 6-year efficacy and safety data from an open-label trial
Source: Arthritis Res Ther. 2019 May 23;21:125. doi: 10.1186/s13075-019-1916-9 (PMC6533709; doi:10.1186/s13075-019-1916-9)
Supplement: Supplementary file 1 — Table S1. Summary of missing data imputation methods for the responder analysis. Table S2. Disease activity and patient-reported outcomes (observed cases). Table S3. Outcomes specific for enthesitis-related arthritis and psoriatic arthritis (observed cases). Table S4. The most frequent TEAEs, excluding infections and injection site reactions (> 5% in any JIA subtype, by System Organ Class). Figure S1. ACR30–100 and JIA inactive disease response rates (OC vs NRI). Additional Tables (A1 to A4) and Figure A1 related to missing data imputation based on patients’ enrolment status, trial period at cut-off date, and reasons for permanent discontinuation. (DOCX 224 kb) [file 13075_2019_1916_MOESM1_ESM.docx]

**Additional file 1**

A hybrid method for missing values imputation was based on patients’ enrolment status, trial period at cut-off date, and reasons for permanent discontinuation, according to the following rules:

| **Table S1. Summary of missing data imputation methods for the responders analysis** | |
| --- | --- |
| **Patients** | **Missing data imputation method** |
| Did not complete CLIPPER | NRI |
| Completed CLIPPER, did not enrol in CLIPPER 2 | LOCF (using final CLIPPER values) |
| Enrolled in CLIPPER 2 and… |  |
| … in active treatment period at cut-off date | OC |
| … in withdrawal or retreatment period at cut-off date, or permanently discontinued for reasons related to pregnancy | LOCF (using final values from Active Treatment Period) |
| … in observational period at cut-off date, or permanently discontinued for reasons NOT related to pregnancy | NRI |
| … had missing values before the amended protocol added efficacy measures | LOCF |
| …went directly to observational period | LOCF |
| …entered withdrawal period or dropped out for reasons related to pregnancy | LOCF |
| …did not enter withdrawal period and had no efficacy data at the time of cut-off | LOCF |
| Abbreviations: LOCF, last observation carried forward; NRI, non-responder imputation; OC, observed cases. | |

| **Table S2. Disease Activity and Patient-Reported Outcomes (Observed Cases)** | | | | | | |
| --- | --- | --- | --- | --- | --- | --- |
| **Measure,**  **Mean**  **(95% CI)** | **CLIPPER** | | **CLIPPER 2** | | | |
|  | **Baseline** | **Month 24** | **Month 36** | **Month 48** | **Month 60** | **Month 72** |
| PtGA  Score | 5.0  (4.6, 5.4)  n=127 | 1.0  (0.7, 1.2)  n=109 | 1.2  (0.8, 1.5)  n=84 | 1.3  (0.9, 1.8)  n=64 | 1.3  (0.9, 1.8)  n=57 | 1.3  (0.9, 1.7)  n=47 |
| PGA  Score | 5.02  (4.7, 5.3)  n=127 | 0.6  (0.5, 0.8)  n=108 | 0.7  (0.5, 0.9)  n=86 | 0.7  (0.5, 0.9)  n=63 | 0.6  (0.4, 0.9)  n=57 | 0.8  (0.5, 1.0)  n=47 |
| CHAQ  Score | 0.80  (0.7, 0.9)  n=127 | 0.2  (0.1, 0.2)  n=108 | 0.2  (0.1, 0.2)  n=62 | 0.1  (0.0, 0.2)  n=37 | 0.2  (0.0, 0.3)  n=22 | 0.2  (0.0, 0.3)  n=16 |
| CRP, mg/L | 8.3  (5.7, 10.8)  n=127 | 2.8  (1.7, 3.8)  n=103 | 3.0  (1.4, 4.5)  n=82 | 2.8  (1.6, 4.1)  n=64 | 2.6  (1.0, 4.2)  n=55 | 1.6  (1.2, 2.0)  n=46 |
| Active joints, n | 6.7  (5.9, 7.6)  n=127 | 0.6  (0.2, 1.0)  n=109 | 0.6  (–0.1, 1.3)  n=69 | 0.5  (0.3, 0.7)  n=64 | 0.4  (0.2, 0.6)  n=56 | 0.4  (0.1, 0.7)  n=46 |
| Joints with  LOM, n | 5.7  (5.0, 6.5)  n=127 | 1.1  (0.5, 1.6)  n=109 | 0.7  (0.1, 1.4)  n=69 | 1.0  (0.6, 1.4)  n=64 | 1.0  (0.5, 1.5)  n=56 | 0.7  (0.3, 1.2)  n=46 |
| JADAS  73 joints score | 17.2  (15.9, 18.4)  n=119 | 2.3  (1.7, 2.9)  n=102 | 2.8  (1.8, 3.7)  n=63 | 2.4  (1.8, 3.1)  n=62 | 2.4  (1.7, 3.0)  n=54 | 2.4  (1.7, 3.2)  n=45 |
| Abbreviations: CHAQ, Childhood Health Assessment Questionnaire; CI, confidence interval; CRP, C-reactive protein; LOM, limitation of motion; PGA, Physician’s Global Assessment; PtGA, patient/Parent Global Assessment; JADAS, Juvenile Arthritis Disease Activity Score. | | | | | | |

| **Table S3. Outcomes specific for enthesitis-related arthritis and psoriatic arthritis (Observed Cases)** | | | | | | |
| --- | --- | --- | --- | --- | --- | --- |
| **Measure,**  **Mean**  **(95% CI)** | **CLIPPER** | | **CLIPPER 2** | | | |
|  | **Baseline** | **Month 24** | **Month 36** | **Month 48** | **Month 60** | **Month 72** |
| **Enthesitis-related arthritis** |  |  |  |  |  |  |
| Back pain, score | 25.9  (16.6, 35.3)  n=37 | 2.4  (0.7, 4.1)  n=30 | 1.6  (0.0, 3.1)  n=21 | 3.8  (-0.1, 7.6)  n=22 | 3.5  (0.9, 6.2)  n=20 | 6.4  (0.9, 11.9)  n=17 |
| Nocturnal back pain, score | 16.4  (7.2, 25.5)  n=38 | 2.2  (0.9, 3.5)  n=30 | 1.6  (0.6, 2.7)  n=21 | 1.4  (0.5, 2.3)  n=22 | 2.6  (0.1, 5.2)  n=20 | 3.0  (0.2, 5.8)  n=17 |
| **Psoriatic arthritis** |  |  |  |  |  |  |
| BSA, % | 9.8  (4.6, 15.0)  n=29 | 1.1  (0.3, 2.0)  n=25 | 1.2  (0.3, 2.0)  n=15 | 1.4  (0.4, 2.3)  n=13 | 2.8  (-0.4, 6.0)  n=13 | 2.2  (0.6, 4.0)  n=11 |
| PGA of Psoriasis, score | 1.8  (1.2, 2.3)  n=29 | 0.5  (0.1, 0.8)  n=25 | 0.8  (0.3, 1.3)  n=14 | 0.7  (0.2, 1.2)  n=13 | 0.9  (0.3, 1.4)  n=14 | 0.9  (0.3, 1.5)  n=11 |
| BSA, body surface area; CI, confidence interval; PGA, Physician’s Global Assessment. | | | | | | |

| **Table S4: The Most Frequent TEAEs, Excluding Infections and Injection Site Reactions (>5% in any JIA Subtype, by System Organ Class)** | | | | |
| --- | --- | --- | --- | --- |
|  | **eoJIA**  **n=60** | **ERA**  **n=38** | **PsA**  **n=29** | **Total**  **N=127** |
| Any TEAE | 49 (81.7) | 31 (81.6) | 22 (75.9) | 102 (80.3) |
| Blood and Lymphatic System Disorders |  |  |  |  |
| - Anemia | 2 (3.3) | 2 (5.3) | 1 (3.4) | 5 (3.9) |
| - Leukopenia | 6 (10.0) | 2 (5.3) | 1 (3.4) | 9 (7.1) |
| - Neutropenia | 4 (6.7) | 0 | 1 (3.4) | 5 (3.9) |
| Ear and Labyrinth Disorders |  |  |  |  |
| - Vertigo | 0 | 1 (2.6) | 2 (6.9) | 3 (2.4) |
| Eye Disorders |  |  |  |  |
| - Uveitis | 3 (5.0) | 2 (5.3) | 2 (6.9) | 7 (5.5) |
| Gastrointestinal Disorders |  |  |  |  |
| - Abdominal pain | 3 (5.0) | 4 (10.5) | 1 (3.4) | 8 (6.3) |
| - Crohn’s disease | 1 (1.7) | 2 (5.3) | 0 | 3 (2.4) |
| - Diarrhea | 3 (5.0) | 6 (15.8) | 1 (3.4) | 10 (7.9) |
| - Gastritis | 1 (1.7) | 2 (5.3) | 0 | 3 (2.4) |
| - Nausea | 6 (10.0) | 2 (5.3) | 0 | 8 (6.3) |
| - Vomiting | 6 (10.0) | 1 (2.6) | 0 | 7 (5.5) |
| General Disorders |  |  |  |  |
| - Asthenia | 0 | 3 (7.9) | 0 | 3 (2.4) |
| - Fatigue | 0 | 3 (7.9) | 0 | 3 (2.4) |
| - Pyrexia | 9 (15.0) | 1 (2.6) | 4 (13.8) | 14 (11.0) |
| Injury, Poisoning, and Procedural Complications |  |  |  |  |
| - Contusion | 3 (5.0) | 3 (7.9) | 0 | 6 (4.7) |
| - Laceration | 0 | 2 (5.3) | 0 | 2 (1.6) |
| Investigations |  |  |  |  |
| - ALT increased | 3 (5.0) | 3 (7.9) | 0 | 6 (4.7) |
| - AST increased | 4 (6.7) | 2 (5.3) | 0 | 6 (4.7) |
| - Hepatic enzyme increased | 2 (3.3) | 1 (2.6) | 2 (6.9) | 5 (3.9) |
| Metabolism and Nutrition Disorders |  |  |  |  |
| - Decreased appetite | 0 | 2 (5.3) | 0 | 2 (1.6) |
| Musculoskeletal and Connective Tissue Disorders |  |  |  |  |
| - Arthralgia | 7 (11.7) | 5 (13.2) | 3 (10.3) | 15 (11.8) |
| - Arthritis | 2 (3.3) | 3 (7.9) | 0 | 5 (3.9) |
| - Back pain | 0 | 1 (2.6) | 3 (10.3) | 4 (3.1) |
| - Juvenile arthritis | 1 (1.7) | 2 (5.3) | 0 | 3 (2.4) |
| - Myalgia | 1 (1.7) | 3 (7.9) | 0 | 4 (3.1) |
| Neoplasms Benign, Malignant, and Unspecified (Including Cysts and Polyps) |  |  |  |  |
| - Skin papilloma | 2 (3.3) | 0 | 2 (6.9) | 4 (3.1) |
| Neoplasms Malignant |  |  |  |  |
| - Hodgkin Lymphoma | 1 (1.7) | 0 | 0 | 1 (0.8) |
| Nervous System Disorders |  |  |  |  |
| - Dizziness | 0 | 2 (5.3) | 1 (3.4) | 3 (2.4) |
| - Syncope | 0 | 3 (7.9) | 0 | 3 (2.4) |
| Psychiatric Disorders |  |  |  |  |
| - ADHD | 0 | 3 (7.9) | 0 | 3 (2.4) |
| Reproductive System and Breast Disorders |  |  |  |  |
| - Dysmenorrhea | 1 (1.7) | 0 | 4 (13.8) | 5 (3.9) |
| Respiratory, Thoracic, and Mediastinal Disorders |  |  |  |  |
| - Cough | 7 (11.7) | 1 (2.6) | 0 | 8 (6.3) |
| - Wheezing | 0 | 2 (5.3) | 0 | 2 (1.6) |
| Skin and Subcutaneous Tissue Disorders |  |  |  |  |
| - Acne | 3 (5.0) | 2 (5.3) | 0 | 5 (3.9) |
| - Psoriasis | 1 (1.7) | 0 | 3 (10.3) | 4 (3.1) |
| ^a^All opportunistic infections were herpes zoster.  ^b^Iridocyclitis, n=4; uveitis, n=3; Crohn’s disease, n=3.  Abbreviations: ADHD, attention deficit/hyperactivity disorder; ALT, alanine aminotransferase; AST, aspartate aminotransferase; TEAE, treatment-emergent adverse event. | | | | |

| **Figure S1. ACR30-100 and JIA Inactive Disease Response Rates (OC vs NRI)** |
| --- |
| 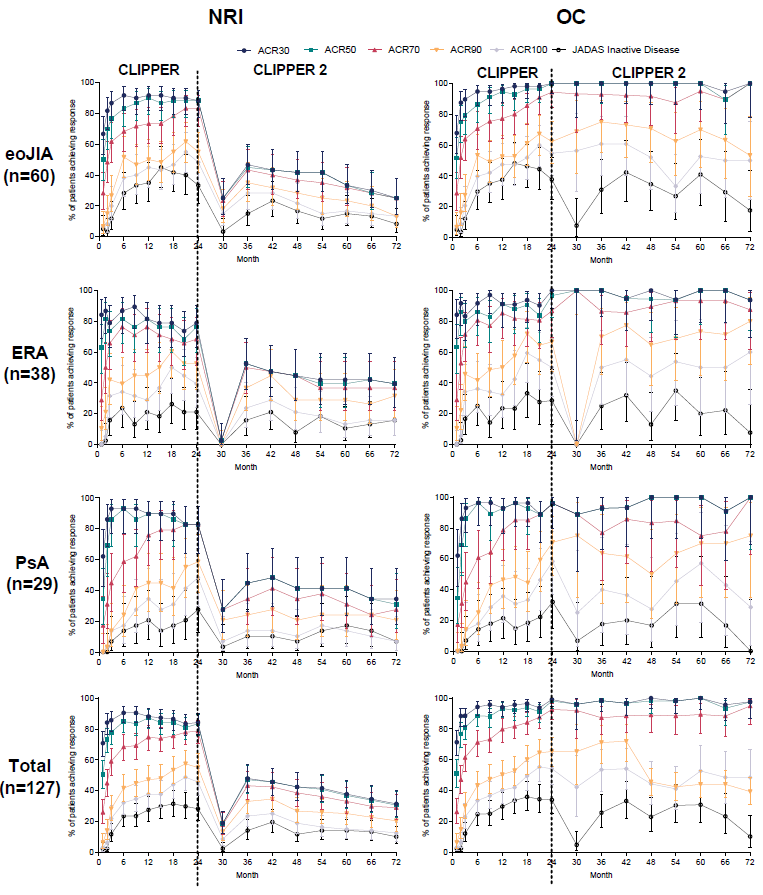 |
| Abbreviations: eoJIA, extended oligoarticular juvenile idiopathic arthritis; ERA, enthesitis-related arthritis; NRI, non-responder imputation; PsA, psoriatic arthritis. OC, observed cases. |
